# Spectrophotometric analysis of purple urine secondary to methylene blue and hydroxocobalamin co-administration

**DOI:** 10.1007/s40620-023-01769-8

**Published:** 2023-08-29

**Authors:** Jeremy Hardin, Henrik Galust, Richard Franklin Clark, Binh Ly, Raymond Theodore Suhandynata

**Affiliations:** 1 Division of Medical Toxicology, Department of Emergency Medicine, UC San Diego Health, 200 W. Arbor Dr. #8676, San Diego, CA 92103 USA; 2 VA San Diego Healthcare System, San Diego, CA USA; 3 San Diego Division, California Poison Control System, San Diego, CA USA; 4 Skaggs School of Pharmacy and Pharmaceutical Sciences, UC San Diego Health, San Diego, CA USA; 5 Department of Pathology, UC San Diego Health, San Diego, CA USA

**Keywords:** Spectrophotometry, Purple urine, Methylene blue, Hydroxocobalamin

## Abstract

**Background:**

The development of purple urine after methylene blue (methylthioninium chloride) and hydroxocobalamin co-administration is a rare clinical entity that has not been fully elucidated. A 47-year-old male presented to the emergency department with hypotension, cyanosis, and depressed mental status. The patient was noted to have profound peripheral and central cyanosis, as well as chocolate-colored arterial blood. He was treated with both methylene blue and hydroxocobalamin and developed purple urine for approximately 1 week.

**Methods:**

Color chromatography was performed by placing the patient’s urine directly onto absorbent filter paper. Urine spectrophotometry was performed utilizing the NanoDrop One/One C UV–Vis Spectrophotometer.

**Results:**

Color chromatography of the urine was demonstrated clear separation of distinct red and blue phases. Urine spectrophotometry demonstrated near perfect overlap between the methylene blue + hydroxocobalamin absorbance spectrum and the patient’s purple urine absorbance spectrum.

**Conclusion:**

Purple urine secondary to methylene blue and hydroxocobalamin co-administration is due to combined urinary excretion of methylene blue (blue) and hydroxocobalamin (red), and not a novel purple metabolite. We anticipate that this is going to be an increasingly common clinical entity as the roles of both hydroxocobalamin and methylene blue expand from toxicologic antidotes to adjunct therapies for vasoplegia, poor cardiac output, and sepsis.

## Introduction

The development of purple urine after methylene blue (methylthioninium chloride) and hydroxocobalamin co-administration is a rare clinical entity. This phenomenon has been previously described but the cause of the purple discoloration has not been fully elucidated. In this study, we identify the cause of purple urine secondary to methylene blue and hydroxocobalamin co-administration by utilizing color chromatography and spectrophotometric analysis.

## Case report

A 47-year-old male presented to the emergency department with hypotension, profound cyanosis, and depressed mental status. Initial vital signs were heart rate 125 beats/minute, blood pressure 75/56 mm Hg, respiratory rate 20 breaths/minute, and oxygen saturation 82% that did not increase with supplemental oxygen. The patient was noted to have profound peripheral and central cyanosis, as well as chocolate colored arterial blood. He was intubated for airway protection and empirically treated with 5 g hydroxocobalamin (Cyanokit, BTG, London, United Kingdom) without clinical improvement. Initial co-oximetry was notable for a methemoglobin level of 66% and PaO2 202 mmHg. Medical toxicology was consulted and the patient received 2 mg/kg methylene blue with rapid clinical improvement. His hemodynamics normalized and cyanosis resolved within one hour, at which point the repeat methemoglobin level was 22% and he was extubated. He endorsed accidentally ingesting “poppers” containing isobutyl nitrite thinking it was a shot of alcohol and remembered rapidly becoming lightheaded before losing consciousness.

**Fig. 1:**
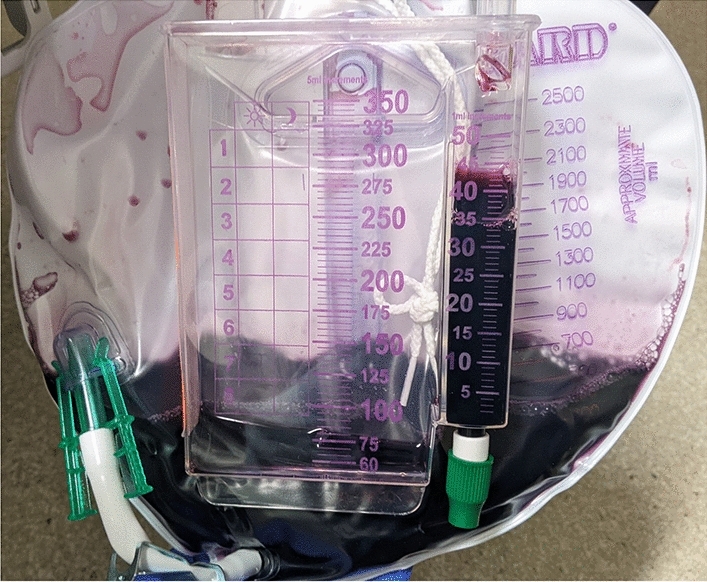
Purple urine discoloration following hydroxocobalamin and methylene blue co-administration

Interestingly, the patient’s urine developed an initial red hue following hydroxocobalamin administration that transitioned to a deep purple following methylene blue administration (Fig. 1). Urinalysis performed at that time demonstrated violet color, negative leukocyte esterase, 2 + nitrite, negative bacteria, and trace blood. The patient denied any dysuria or urinary complaints, and left on hospital day two via self-directed discharge. On phone follow up one month later he was doing well and stated the purple discoloration of his urine persisted for one week after leaving the hospital.

## Discussion

Purple urine is a rare clinical entity with multiple potential etiologies. In the setting of isolated methylene blue administration purple urine raises the concern for acute hemolytic anemia secondary to glucose-6-phosphate dehydrogenase deficiency [1]. Dietary causes of purple urine include “beeturia” secondary to beet ingestion and subsequent betacyanin pigment excretion [2]. Excess urinary porphyrins (which share a similar structure with hydroxocobalamin) have also been implicated. Purple urine can also be generated ex-vivo with the sodium dithionite paraquat detection assay [3].

Purple urine bag syndrome is a distinct clinical entity that was first described in 1978 [4]. It is a sign of severe urinary tract infection, particularly in elderly individuals with chronic indwelling urinary catheters colonized by *Escherichia coli*, *Proteus mirabilis*, or *Klebsiella pneumoniae* [5]. The mechanism is well understood and involves the metabolism of tryptophan to indole by intestinal bacteria, conjugation to indoxyl sulfate in the liver, and final metabolism to indirubin (red) and indigo (blue) by bacterial phosphatase leading to purple colored urine [5]. Purple urine bag syndrome was not present in this case as the patient did not have known risk factors or evidence of urinary tract infection on urinalysis (2 + nitrites were attributed to ingestion of isobutyl nitrite).

Hydroxocobalamin is indicated for the treatment of acute cyanide toxicity, and red discoloration of mucous membranes, serum, and urine is a well described side effect of therapy [6]. A study of 102 volunteers demonstrated that hydroxocobalamin-induced red chromaturia was universal [7]. Likewise, methylene blue is indicated for the treatment of methemoglobinemia and is well described to cause blue/green discoloration of the urine [8]. Both compounds are predominantly renally excreted with 29% of methylene blue and 26% of hydroxocobalamin excreted at 24 h post-administration [9, 10]. Hydroxocobalamin and methylene blue are also used off-label for the treatment of vasoplegia, poor cardiac output, and distributive shock [11]. Early research for this off-label indication is promising, and we anticipate increased use of both medications in the future, particularly in the intensive care unit setting [11–14].

At the time of this report, there has only been one other study which describes the rare iatrogenic clinical entity of purple urine secondary to hydroxocobalamin and methylene blue co-administration [15]. Aklilu et al. speculated that there did not seem to be any known pathway that would generate a purple metabolite, and that the urine coloration was likely the result of the combination of the blue and red colors from methylene blue and hydroxocobalamin, respectively [15]. Our analysis set out to definitively answer this question.

## Results

### Urine color chromatography

**Fig. 2:**
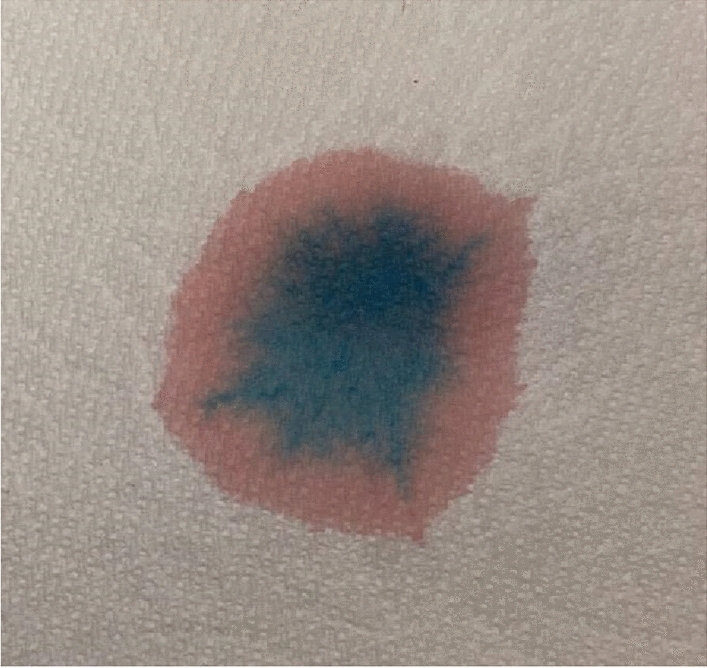
Color chromatography demonstrating separation of red and blue components

Color chromatography was initially performed by placing 3 mL of the patient’s urine directly onto absorbent filter paper. After five minutes clear separation of distinct red and blue phases was observed (Fig. 2). This finding supports the hypothesis that the purple color is a result of two separate compounds and prompted definitive testing with a scanning spectrophotometer.

### Urine spectrophotometry

**Fig. 3:**
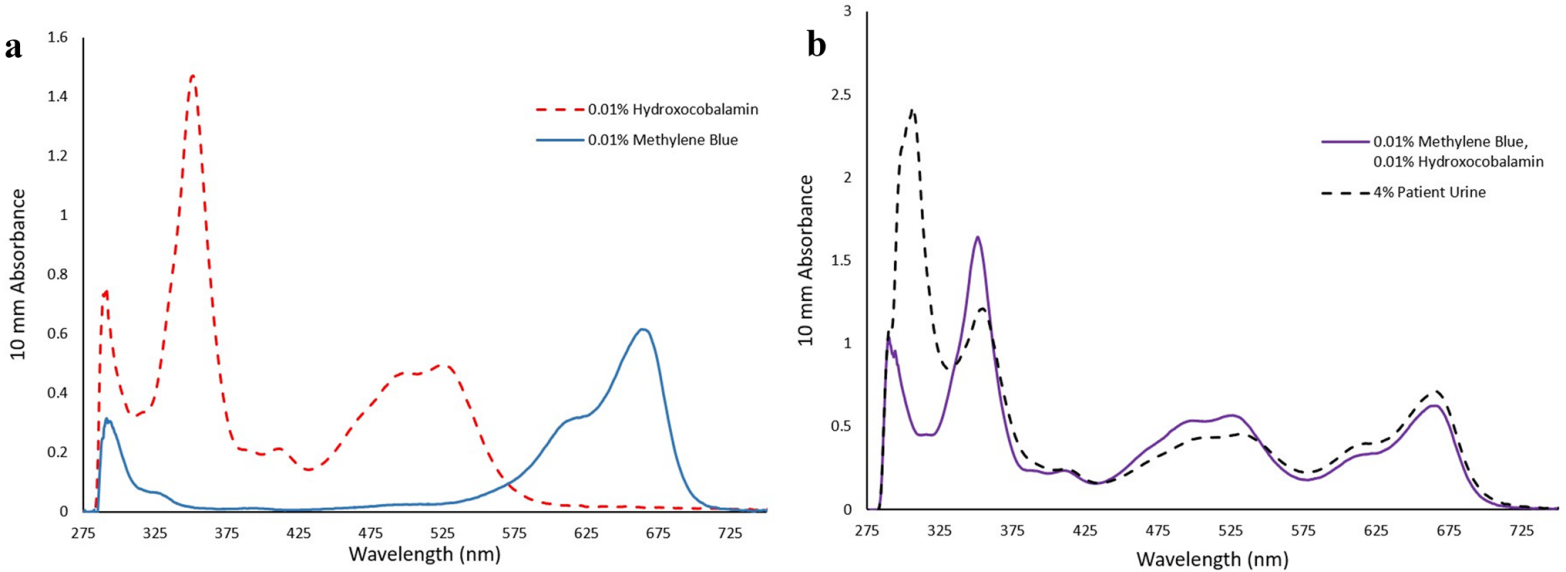
Absorbance spectrum of **a** 0.01% methylene blue and 0.01% hydroxocobalamin standards **b** 4% purple urine and the combined mixture of 0.01% methylene blue and 0.01% hydroxocobalamin

Urine spectrophotometry was performed utilizing the NanoDrop One/One C UV–Vis Spectrophotometer (Thermo Fisher Scientific) that was set to begin scanning at 190 nm and end at 850 nm with a step size of 0.5 nm. Samples were analyzed in a 1 mL plastic cuvette with a 1 cm pathlength. Standard solutions of 0.01% hydroxocobalamin (Sigma-Aldrich, St. Louis, MO, USA) and 0.01% methylene blue (Biopharm, San Mateo, CA, USA) were prepared in 18-megaohm deionized water and analyzed separately to establish their absorption spectrum (Fig. 3a). Patient urine was diluted using 18-megaohm deionized water. The methylene blue and hydroxocobalamin standard solutions were then mixed and the absorbance spectrum of the mixture was obtained and compared to the absorbance spectrum of a 4% solution of the patient’s urine (Fig. 3b). Overlap between the methylene blue + hydroxocobalamin absorbance spectrum and the patient’s purple urine absorbance spectrum was nearly perfect. Maximum absorbance peaks were observed at 290 nm, 351 nm, 526 nm, and 665 nm for the methylene blue + hydroxocobalamin mixture. Maximum absorbance peaks were observed at 306 nm, 355 nm, 530 nm, and 665.5 nm for the 4% solution of the patient’s urine.

## Conclusion

Purple urine secondary to methylene blue and hydroxocobalamin co-administration is due to the combined urinary excretion of methylene blue (blue) and hydroxocobalamin (red), and not a novel purple metabolite. This finding reportedly lasted for one week in our patient—a duration that is consistent with the only other reported case [15]. Unlike purple urine bag syndrome, this clinical entity is benign and does not necessitate further evaluation or treatment. We anticipate that this is going to be an increasingly common clinical entity as the roles of both hydroxocobalamin and methylene blue expand from toxicologic antidotes to adjunct therapies for vasoplegia, poor cardiac output, and shock [11–14].
